# Appropriate Number of Treatment Sessions in Virtual Reality-Based Individual Cognitive Behavioral Therapy for Social Anxiety Disorder

**DOI:** 10.3390/jcm10050915

**Published:** 2021-02-26

**Authors:** Hyu Seok Jeong, Jee Hyun Lee, Hesun Erin Kim, Jae-Jin Kim

**Affiliations:** 1 Department of Psychiatry, Gangnam Severance Hospital, Yonsei University College of Medicine, Seoul 06273, Korea; HYOSUC@yuhs.ac; 2 Institute of Behavioral Sciences in Medicine, Yonsei University College of Medicine, Seoul 03722, Korea; erinkim791@yuhs.ac; 3 Department of Social Work and Services, Gangnam Severance Hospital, Yonsei University College of Medicine, Seoul 06273, Korea; LECIELLA@yuhs.ac

**Keywords:** social anxiety disorder, virtual reality, individual cognitive behavioral therapy, exposure therapy, number of sessions

## Abstract

Virtual reality (VR) was introduced to maximize the effect of cognitive behavioral therapy (CBT) by efficiently performing exposure therapy. The purpose of this study was to find out whether VR-based individual CBT with relatively few treatment sessions is effective in improving social anxiety disorder (SAD). This therapy was applied to 115 patients with SAD who were retrospectively classified into 43 patients who completed the nine or 10 sessions normally (normal termination group), 52 patients who finished the sessions early (early termination group), and 20 patients who had extended the sessions (session extension group). The Brief Fear of Negative Evaluation Scale (BFNE) scores tended to decrease in all groups as the session progressed, and the slope of decrease was the steepest in the early termination group and the least steep in the session extension group. Severity of social anxiety in the last session and symptom reduction rate showed no significant group difference. Our findings suggest that short-term VR-based individual CBT of nine to 10 sessions may be effective. When the therapeutic effect is insufficient during this period, the additional benefit may be minimal if the session is simply extended. The improvement in the early termination group suggests that even shorter sessions of five or six can also be effective.

## 1. Introduction

Social anxiety disorder (SAD) characterized by a marked fear about social situations, and avoidance of social stimuli [1] is a common psychiatric disorder with lifetime prevalence between 7% and 13% [2,3]. Cognitive behavioral therapy (CBT) has been used as a major tool for the treatment of SAD [4,5]. Among the techniques of CBT, exposure therapy is often used to treat a variety of anxiety disorders [6,7]. In particular, in vivo exposure to social situations has been reported to be an effective way to alleviate social anxiety and overcome social avoidance [8,9]. For example, Haug et al. [8] reported that between 24 weeks of treatment and 52 weeks of follow-up evaluation, the Clinical Global Impression—Social Phobia overall severity score decreased by 0.45 in the in vivo exposure group, compared to 0.25 in the placebo group.

However, in vivo exposure can be impractical to use in clinical practice due to several issues, such as cost, time consumption, audience recruitment, and control management. Patients with SAD can avoid experiencing such real-world situations because of their symptoms. Virtual reality (VR) exposure can be an alternative to in vivo exposure in that VR can induce emotional responses similar to reality and its application is acceptable to patients and produces a good therapeutic effect [10]. As a result of these advantages, VR exposure therapy (VRET) is being used more and more often in the clinical field to treat various mental disorders [11,12]. In particular, VRET has been reported to be as effective as traditional CBT in treating public speaking anxiety [13,14] and even generalized SAD [15]. A recent controlled trial for patients with SAD showed that VRET was more effective at post-treatment and more practical for therapists than in vivo exposure therapy [16]. A recent meta-analysis study suggested that the efficacy of VRET for SAD continued over the long-term follow-up period with an effect size (Hedges′ g) of −0.86 at post-intervention, –1.03 at three months, −1.14 at six months, and −0.74 at 12 months [17]. Our research team also reported that the use of VR exposure technology was effective in reducing social anxiety even in a self-training environment; the Liebowitz Social Anxiety Scale (LSAS) score decreased 19.8% in patients with social anxiety disorder [18].

In addition to exposure therapy, other forms of CBT have also been reported to be effective in treating SAD, examples of which include group therapy [19], individual cognitive therapy [20], intensive group cognitive therapy [21], social skills training [22], relaxation training [23], and mindfulness-based stress reduction [24]. Meta-analytic reviews have reported that combining two or more of these types of CBT is also useful for treating SAD, and both individual and group formats are effective [25,26]. In addition, in order to maintain the therapeutic effect for a sufficiently long period after CBT, it is necessary to weaken the negative beliefs of patients with SAD, and for this, cognitive restructuring through intensive cognitive therapy may be essential [27]. Taken together, it may be possible to treat patients with SAD only with VRET, but combining this with other forms of CBT is likely to improve treatment effectiveness. For example, VR-based individual CBT may include cognitive restructuring and relaxation training as well as VRET.

There is no doubt about the usefulness of CBT as such, but there are still no definitive guidelines on how many treatment sessions should be conducted to effectively treat SAD. Previous studies have reported that long-term treatment of 14 or 16 sessions, once a week, was effective [16,28], while short-term treatment of eight sessions was also effective [13]. Our research team reported that eight intensive exposure sessions for 2 weeks were effective enough to reduce social anxiety [18]. Due to the diversity of the treatment session composition of previous studies, it has not been established how many treatment sessions are appropriate even for VR-based individual CBT that maximizes the effect of CBT by efficiently performing exposure therapy.

The purpose of this study was to find out whether VR-based individual CBT with relatively few treatment sessions of nine or 10 times is effective in improving SAD. For this purpose, we performed a retrospective analysis of the data from patients who received VR-based individual CBT for the treatment of SAD, the core of which was to compare psychological measures among the group who ended earlier than eight sessions, the group who normally ended nine or 10 sessions, and the group who conducted longer than 11 sessions. We hypothesized that nine or 10 sessions of therapy would be sufficient to alleviate social anxiety, and in some cases, even short-term therapy of eight or fewer sessions could achieve the desired outcome, whereas there would be little additional benefit that could be gained from long-term therapy of longer than 11 sessions.

## 2. Methods

### 2.1. Participants

Among patients with SAD who visited the department of psychiatry in Gangnam Severance Hospital, 115 patients who participated in VR-based CBT were the targets of the retrospective analysis. Psychiatric diagnosis was based on the Mini-International Neuropsychiatric Interview (MINI) [29], and exclusion criteria included other significant psychiatric illnesses except SAD, substance abuse, any neurological or serious physical disorders, and a pregnancy. This study was approved by the institutional review board of Gangnam Severance Hospital (3-2020-0431).

### 2.2. Procedure

All patients participated in VR-based CBT for nine or 10 weeks in one session per week. These sessions were conducted one-to-one between a therapist skilled in CBT (J.H.L.) and a patient in the VR clinic of Gangnam Severance Hospital. VR-based CBT included cognitive restructuring training, relaxation training, and VRET. Cognitive restructuring training was to address the frequency and strength of beliefs to reduce negative self-defeating thoughts and increase positive well-being-enhancing thoughts [30]. Relaxation training consisted of diaphragmatic breathing and progressive muscle relaxation [31]. VRET was conducted using the desktop-based program or the mobile version consisting of three environments, in which 12 situations and 36 topics were provided with four different levels of difficulty in a way that the number of virtual audience appearing increased (see the sample videos: https://youtu.be/LxfSPaSJSTE accessed on 1 December 2020). The program featured an automatic monitoring system of the participants′ eye movement, speaking time, and heart rate for immediate feedback. A further description of VRET is detailed in a previous paper [18].

During the VR-based CBT period, the progression of sessions was in the following sequence and contents: Session 1, orientation and psychoeducation for SAD; Session 2, cognitive restructuring training (recognizing automatic thinking and cognitive errors); Session 3, cognitive restructuring training (attacking cognitive errors and deriving rational responses) and VRET (level 1, conversation with some people); Session 4, cognitive restructuring training (correcting irrational beliefs and becoming a self-therapist) and VRET (level 2, presentation in front of a small audience size); Session 5, relaxation training (diaphragmatic breathing) and VRET (level 3, presentation in front of a moderate audience size); Session 6, relaxation training (progressive muscle relaxation) and VRET (level 3, presentation in front of a moderate audience size); Session 7, VRET (level 4, presentation in front of a large audience size); Session 8, VRET (level 4, presentation in front of a large audience size); Session 9, VRET (special level, presentation in front of evaluators); and Session 10, VRET (special level, presentation in front of evaluators).

In principle, this training schedule was applied to all participants, but it was flexibly applied depending on the participants′ symptoms and learning level. Ten sessions were the default, but when nine sessions yielded sufficient treatment effects, the VR-based CBT was terminated normally under the agreement of the therapist and participant. If it was judged that sufficient therapeutic effect was not obtained by the 9th session, the VR-based CBT could be extended to more than 11 sessions with the agreement of the two. In these extended sessions, the contents of training were basically re-learning over and over again what was included up to the 10th session, and the termination was made between the 11th and 20th sessions by the agreement between the two.

### 2.3. Measurements

Prior to the beginning of the first session, participants completed a set of questionnaires. The first one was the Brief Fear of Negative Evaluation Scale (BFNE), which is a 12-item 5-point Likert scale (1–5) to assess anxiety with perceived negative evaluation [32]. The second one was the LSAS, which is a 24-item 4-point Likert scale (0–3), assessing the level of social anxiety and the avoidance of different social situations [33]. The third one was the Social Phobia Scale (SPS), which is a 20-item 5-point Likert scale (0–4), assessing fears of being scrutinized during routine activities [34]. The fourth one was the Social Interaction Anxiety Scale (SIAS), which is a 20-item 5-point Likert scale (0–4), measuring complementary aspects of social phobia [34]. These measures were reevaluated after the last session. Given that the BFNE was sensitive to treatment-related change and thus useful as an outcome measure in SAD [35], the BFNE was repeatedly evaluated in each session.

### 2.4. Statistical Analysis

The participants were classified into the following three groups according to the number of sessions completed. The participants who did not reach the 9th session, the criterion for normal termination, were referred to as the early termination group. The participants who completed the 9th or 10th session were referred to as the normal termination group. The participants who extended the session more than 11 times by agreement with the therapist were referred to as the session extension group.

Demographic variables and psychological measures at baseline were compared among the three groups using analysis of variance (ANOVA) or chi-squared test. The significance of changes in the BFNE score according to the progress of treatment sessions and their difference among the three groups were analyzed using the linear mixed model (specifically, random intercept model) analysis. For this analysis, after missing data were inputted using the last observation carried forward (LOCF) method, the estimated BFNE scores were counted in all groups and compared using ANOVA. Independent variables as possible predictors for the linear mixed models included gender, age, education years, job status, marital status, medication status, group, number of sessions, and group-by-session interaction. In the normal termination and session extension groups, the changes before and after the therapy of the scale scores other than the BFNE score were analyzed using ANOVA. For all scale scores, the symptom reduction rate was calculated by the formula of [(pretreatment score − posttreatment score) ÷ pretreatment score] × 100%, and the group difference in this reduction rate was assessed using ANOVA or Student t-tests. For all the ANOVAs described above, post-hoc tests for significant results were performed using independent or paired *t*-tests. The significance level in all analyses was set at *p* < 0.05, and Bonferroni correction was additionally applied to post-hoc tests. All statistical analyses were conducted using SPSS statistics, v. 25.0 (IBM, Armonk, NY, USA), SAS version 9.4 (SAS Institute Inc., Cary, NC, USA), or R package version 4.0.3 (http://www.R-project.org accessed on 15 December 2020).

## 3. Results

### 3.1. Participant Characteristics

Among 115 participants, 52 were in the early termination group, 43 were in the normal termination group, and 20 were in the session extension group. The total number of sessions was 5.2 ± 1.9 in the early termination group, 9.4 ± 0.9 in the normal termination group, and 13.5 ± 2.7 in the session extension group. Demographic variables and measures of social anxiety in these three groups before VR-based CBT are given in Table 1. There were no significant group differences in demographic information. There were also no significant differences in the BFNE, LSAS-avoidance, and SIAS scores among the three groups, whereas the LSAS-anxiety and SPS scores showed a significant group difference (*F*_2112_ = 6.19, *p* = 0.003; *F*_281_ = 5.35, *p* = 0.007, respectively). Post-hoc tests showed that both the LSAS-anxiety and SPS scores were significantly higher in the session extension group than in the early and normal termination groups (*t*_112_ = 3.44, *p* = 0.003 and *t*_112_ = 2.95, *p* = 0.012; *t*_81_ = 3.05, *p* = 0.009 and *t*_81_ = 2.98, *p* = 0.012; respectively), whereas those scores did not significantly differ between the early and normal termination groups.

### 3.2. Changes in the BFNE Scores According to Session Progress

**Figure 1:**
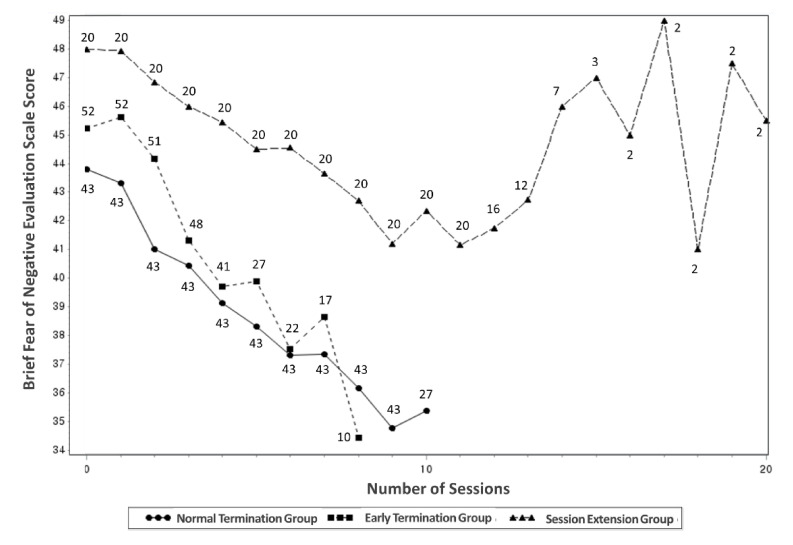
Plots of the mean scores of Brief Fear of Negative Evaluation Scale and the number of participants in each session among the early termination, normal termination, and session extension groups.

**Figure 2:**
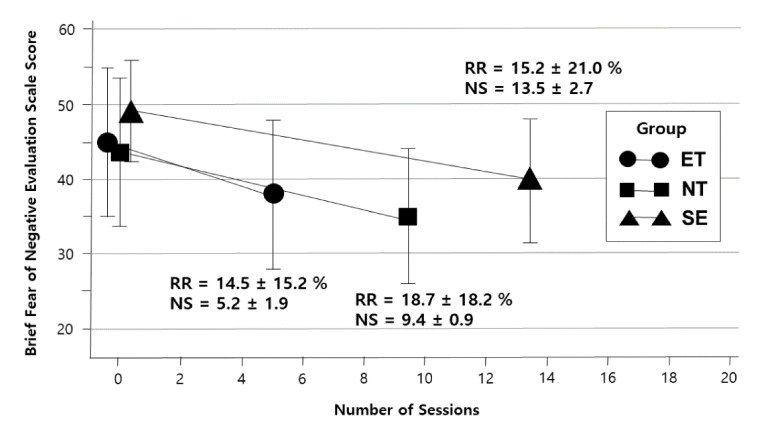
Group-specific changes in the Brief Fear of Negative Evaluation Scale scores for each participant from baseline to the last session. The number of sessions (NS) varied depending on the participants from one to eight in the early termination group (ET), nine to ten in the normal termination group (NT), and 11 to 20 in the session extension group (SE). Reduction rate (RR) = [(score at baseline − score in the last session)/score at baseline] × 100%.

Figure 1 shows plots of the mean BFNE scores and the number of participants in each session and each group. Considering that the number of sessions completed in each group varied depending on the participants, Figure 2 presents changes in the mean BFNE scores from baseline to the last session. The scores in the last session did not significantly differ among the early termination, normal termination group, and session extension groups (38.5 ± 10.2, 35.0 ± 9.5, and 40.0 ± 8.1, respectively; *F*_2112_ = 2.18, *p* = 0.097). The reduction rate of the BFNE score also showed no significant group difference (14.5 ± 15.2 %, 18.7 ± 18.2%, and 15.2 ± 21.0%, respectively; *F*_2,112_ = 1.76, *p* = 0.477).

Table 2 presents the estimated mean BFNE scores at baseline and in each session, which were obtained using the LOCF method, and it shows results from the comparison among the three groups using ANOVA. From baseline to the second session, the estimated scores showed no group difference. By the third session, the scores were significantly higher in the session extension group than in the normal termination group. From the 4th session to the 10th session, they were significantly higher in the session extension group than in the early and normal termination groups. The scores did not differ between the early and normal termination groups in all sessions. Based on these estimated mean BFNE scores, the expected reduction rate when the 10th session was completed was 21.9% in the normal termination group, rather high at 28% in the early intervention group, and only 13.9% in the session extension group.

In the linear mixed models exploring associations of possible predictors with fear of negative evaluation, none of the demographic variables turned out to be significant predictors. Group was also revealed non-significant (*F*_2862_ = 2.41, *p* = 0.091), but the number of sessions and group-by-session interaction effect were significant (*F*_1862_ = 301.77, *p* < 0.0001; *F*_2862_ = 8.84, *p* = 0.0002, respectively). Post-hoc tests showed that all of the early termination, normal termination, and session extension groups showed significant decreases in the BFNE scores according to session progress (slope [standard error]: −1.31 [0.12], *p* < 0.0001; −0.95 [0.07], *p* < 0.0001; and −0.67 [0.09], *p* < 0.0001, respectively). The decrease in the scores was significantly steeper with a difference in slope of 0.36 [0.14] in the early termination group than in the normal termination group (*p* = 0.011), with a difference in slope of 0.29 [0.12] in the normal termination group than in the session extension group (*p* = 0.015), and with a difference in slope of 0.64 [0.15] in the early termination group than in the session extension group (*p* < 0.0001).

### 3.3. Changes in Other Psychological Measures before and after VR-Based CBT

Table 3 shows the psychological scale scores at baseline and in the last session in the normal termination and session extension groups. Since participants in the early termination group did not terminate VR-based CBT in agreement with the therapist, these scales, evaluated at termination, were not obtained, and thus, they were excluded in this table. The main effect of group was shown in the LSAS-anxiety, SPS, and SIAS scores (*F*_1107_ = 11.27, *p* = 0.001; *F*_192_ = 7.43, *p* = 0.008; *F*_191_ = 5.67, *p* = 0.019) but not in the LSAS-avoidance score. Post-hoc t-tests revealed that all of these three scale scores showing the group effect were significantly higher in the session extension group than in the normal termination group (all: *p* < 0.05). The main effect of time was shown in all scale scores (LSAS-anxiety: *F*_1107_ = 16.3, *p* < 0.001, LSAS-avoidance: *F*_1107_ = 8.86, *p* = 0.004, SPS: *F*_192_ = 20.48, *p* < 0.001, and SIAS: *F*_191_ = 19.86, *p* < 0.001, respectively). Post-hoc t-tests revealed that the scores in the last session were significantly lower than those at baseline in all of the four scales (all: *p* < 0.01). The group × time interaction effect was not found in all scale scores, and the reduction rates of all scale scores did not significantly differ between the two groups.

## 4. Discussion

The core issue of this study was the appropriate number of sessions when performing VR-based CBT in patients with SAD. Although it has been repeatedly reported that the treatment effect of VR-based CBT is excellent in SAD, the number of treatment sessions applied for each report varies considerably from four to 16 [36]. VR-based CBT is superior to conventional CBT in terms of cost and effort of delivering the treatment [37], yet there is no controlled study on whether the application of VR to patients with SAD contributes to the reduction of the number of treatment sessions.

Since our VR-based CBT was based on nine to 10 sessions, the patients who completed these sessions were defined as the normal termination group. Given that different cut-offs, from 20 to 50%, have been used in the literature for the definition of response [38], when 20% is considered the minimal response, most of our study′s scale scores appear to have reached the minimal level of improvement through these numbers of sessions. These numbers correspond to a study by Kampmann et al. (2016) [15], reporting the results after performing 10 sessions of VR-based CBT, in which the symptom reduction rate was about 12.2% on the BFNE and 23.6% on the LSAS. Our results showed that this reduction rate was relatively high on the BFNE (21.9%) and slightly low on the LSAS (19.5%). This is believed to be because our therapy was not only focused on exposure therapy using VR but also included cognitive restructuring and relaxation training that did not use VR.

In our VR-based CBT, there were patients who participated in sessions of 11 or more (average 13.5) without terminating normally in the 9th or 10th session, which was designated as the session extension group. These patients showed that all scale scores in the last session decreased to the extent that there was no statistical difference from those of the normal termination group. However, the slopes representing the rate of decline in the BFNE score indicated that this group was the least steep among the three groups, suggesting that they required more sessions to achieve a similar degree of therapeutic effect as other groups of patients. This inefficiency is likely to be related to their severe symptoms before the start of treatment, especially with the LSAS-anxiety and SPS scores significantly being higher than those of the other groups. In general, when predicting the therapeutic effect of psychotherapy, the baseline symptom severity has been regarded as a reliable predictor [39]. Even in SAD, the severity of baseline social anxiety was found to be one of the significant predictors of poor outcome [40].

At the current research level, it is difficult to determine whether long-term treatment of 11 or more sessions is necessary. The answer to this question cannot be found in the previous studies that analyzed the effects of VR-based CBT consisting of 12 or more sessions [14,16,41]. This is because all of them did not report any detailed changes in the scale scores after session 11, only the pre- and post-treatment scale scores. In fact, patients in our session extension group originally started with the goal of normal termination but felt that the treatment effect was insufficient and agreed with the therapist to extend the sessions. This feeling is reflected in the result that the symptom reduction rate calculated based on the estimated BFNE score at the 10th session was only 13.9% in this group. However, even though the therapy was extended, the reduction rate calculated based on the actual BFNE score at the last session was 15.2%, indicating that, as our hypothesis, there was little benefit obtained by adding the sessions. In light of these findings, it may not be recommended to simply extend the treatment sessions based on a patient′s request to obtain more therapeutic effects rather than the scheduled sessions from the initial plan. Instead, it may be desirable to add other treatment modalities for this class of patients.

Meanwhile, patients in the early termination group participated only in eight sessions or less in our VR-based CBT and did not go through a normal termination process. Thus, it was not possible to determine why they gave up treatment early. However, while their personal circumstances or disappointment with the degree of improvement may be the reason, they may have made such a decision because they thought that the treatment was remarkably effective and they did not need further treatment. The possibility for the latter seems to be well reflected by the changes in the BFNE scores of this group. They completed an average of 5.2 sessions, showing an average reduction rate of 14.5% in their last session, which was the lowest among the three groups but not statistically different. Therefore, short-term treatments of five to six sessions may be suitable for rapid responders. This is supported by a previous report that only four sessions of treatment were effective in reducing public speaking anxiety [42]. There is a report that VRET consisting of eight sessions was effective for treating social anxiety, and the therapeutic effect continued even after one year [11]. However, in our analysis, as it was calculated that their expected reduction rate based on the estimated BFNE score could have risen to 28.4% if it had reached normal termination, it seems that the treatment schedule of nine to 10 sessions rather than early termination may help maximize the treatment effect. Meanwhile, the findings in this early termination group can have an important economic implication. If a satisfactory therapeutic effect can be achieved with a small number of VRET sessions, patients will be able to receive treatment at a lower cost. Accordingly, the number of patients participating in the treatment may increase, and hospitals may increase the related investment.

Our results should be interpreted within the context of the study′s limitations. In this study, based on the data of patients with SAD who had already received VR-based CBT, the analysis was conducted in groups that were retrospectively classified according to specific criteria, and thus the number of sessions was too diverse and the same evaluation was not made between groups. For the same reason, demographic variables such as duration of illness or economic status that could affect outcomes were not included in the analysis. Therefore, a prospective randomized controlled study is needed to more accurately evaluate the effect of the number of sessions and the various demographic variables. In addition, the most important scale analyzed in this study was the BFNE measured in each session. This scale focuses on the cognitive aspects of patients with SAD and thus may not reflect patients′ social anxiety and avoidance linearly.

## 5. Conclusions

VR technology benefits treatment outcomes not only for the cost and effort of delivering treatment but also for the effectiveness of treatment. In this retrospective study for an issue of how many sessions are appropriate when performing VR-based CBT, data related to the severity of social anxiety obtained from patients with SAD, who received the therapy, were analyzed focusing on the differences among the three groups, such as the normal termination, early termination, and session extension groups. In the results, fear of negative evaluation tended to decrease in all groups as the session progressed, and the slope of decrease was the steepest in the early termination group and the least steep in the session extension group. The BFNE scores in the last session and the reduction rates of the scores showed no significant group difference. Our results suggest that short-term VR-based individual CBT of nine to 10 sessions may be effective in the treatment of SAD, and that patients with insufficient therapeutic effect during this period are difficult to obtain additional benefits even if the treatment is continued for an extended period. In view of the improvement in patients who finished earlier than normal termination, even shorter sessions of five or six could be effective in treating SAD. A further randomized controlled study is needed to assess a difference in effectiveness depending on the number of sessions more accurately.

## Figures and Tables

**Table 1:** Demographic variables and psychological measures of the early termination, normal termination, and session extension groups before virtual reality-based cognitive behavioral therapy.

	Early Termination Group (n = 52)	Normal Termination Group (n = 43)	Session Extension Group (n = 20)	*χ* ^2^ *or F*	*p*
Gender, male/female	28/24	32/11	15/5	5.41	0.067
Age, years	34.3 ± 13.3	31.2 ± 15.5	27.6 ± 10.7	1.81	0.169
Education level, years *	14.2 ± 3.2	13.0 ± 3.4	13.5 ± 3.1	1.60	0.207
Job status, employed/unemployed	22/30	13/30	7/13	1.51	0.471
Marital status, single/married/divorced	29/20/3	29/12/2	15/5/0	3.33	0.504
Medication, medicated/unmedicated	28/24	23/20	15/5	3.07	0.215
BFNE	45.2 ± 9.7	43.8 ± 10.4	48.0 ± 6.4	2.07	0.264
LSAS, anxiety	34.1 ± 14.6	35.5 ± 13.9	46.6 ± 11.4	6.19	0.003
LSAS, avoidance	27.7 ± 15.7	31.0 ± 14.8	34.6 ± 10.8	1.70	0.187
SPS	32.2 ± 18.5	32.4 ± 18.0	49.2 ± 17.6	5.35	0.007
SIAS	43.9 ± 21.7	46.6 ± 15.9	56.1 ± 12.7	3.62	0.101

Values are the number of participants for Chi-squared test or mean ± standard deviation for ANOVA. * Education level refers to the number of years actually attended school. Abbreviation: BFNE, Brief Fear of Negative Evaluation Scale; LSAS, Liebowitz Social Anxiety Scale; SPS, Social Phobia Scale; SIAS, Social Interaction Anxiety Scale.

**Table 2:** Changes in the estimated mean of the Brief Fear of Negative Evaluation Scale scores according to session progress and group comparisons in each session.

Session Number	Early Termination Group (n = 52)	Normal Termination Group (n = 43)	Session Extension Group (n = 20)	*p*	*Post-hoc*
Baseline	46.0 (1.2)	43.3 (1.3)	48.2 (1.9)	0.089	
1	44.7 (1.2)	42.4 (1.3)	47.5 (1.9)	0.074	
2	43.4 (1.1)	41.4 (1.3)	46.8 (1.8)	0.057	
3	42.1 (1.1)	40.5 (1.2)	46.2 (1.8)	0.041	ET = NT, ET = SE, NT < SE
4	40.8 (1.2)	39.5 (1.2)	45.5 (1.8)	0.028	ET = NT, ET = SE, NT < SE
5	39.4 (1.2)	38.6 (1.2)	44.8 (1.8)	0.019	ET = NT, ET < SE, NT < SE
6	38.1 (1.2)	37.6 (1.2)	44.2 (1.8)	0.012	ET = NT, ET < SE, NT < SE
7	36.8 (1.3)	36.7 (1.3)	43.5 (1.8)	0.008	ET = NT, ET < SE, NT < SE
8	35.5 (1.3)	35.7 (1.3)	42.8 (1.8)	0.005	ET = NT, ET < SE, NT < SE
9	34.2 (1.4)	34.8 (1.3)	42.2 (1.9)	0.004	ET = NT, ET < SE, NT < SE
10	32.9 (1.5)	33.8 (1.3)	41.5 (1.9)	0.003	ET = NT, ET < SE, NT < SE

Values are mean (standard error). Abbreviation: ET, early termination group; NT, normal termination group; SE, session extension group.

**Table 3:** Changes in the psychological scale scores in the last session compared with the baseline scores in the normal termination and session extension groups.

Scale	Normal Termination Group				Session Extension Group				Reduction Rate		Group × Time Interaction	
	Baseline	Last	*p* ^a^	Reduction Rate, %	Baseline	Last	*p* ^a^	Reduction Rate, %	*t* ^b^	*p*	*F* ^c^	*p*
LSAS, anxiety	35.5 ± 13.9	27.0 ± 12.8	0.000	19.5 ± 25.0	46.6 ± 11.4	33.7 ± 11.4	0.002	25.1 ± 26.6	−0.70	0.486	0.64	0.424
LSAS, avoidance	31.0 ± 14.8	23.5 ± 13.7	0.001	13.1 ± 37.9	34.6 ± 10.8	26.0 ± 7.7	0.009	20.7 ± 31.7	−0.67	0.507	0.04	0.848
SPS	32.4 ± 18.0	22.8 ± 15.6	0.001	22.9 ± 41.2	49.2 ± 17.6	25.8 ± 13.9	0.000	43.4 ± 23.4	−1.59	0.121	3.58	0.062
SIAS	46.6 ± 15.9	32.9 ± 15.6	0.000	23.2 ± 26.9	56.0 ± 12.7	39.6 ± 14.5	0.006	25.8 ± 28.4	−0.26	0.796	0.17	0.680

Values are mean ± standard deviation. Abbreviation: LSAS, Liebowitz Social Anxiety Scale; SPS, Social Phobia Scale; SIAS, Social Interaction Anxiety Scale. ^a^ Paired *t*-test, ^b^ Student *t*-test, ^c^ ANOVA.

## Data Availability

The data presented in this study are available on request from the corresponding author. The data are not publicly available due to ethical restrictions.
